# The OPERA trial: a protocol for the process evaluation of a randomised trial of an exercise intervention for older people in residential and nursing accommodation

**DOI:** 10.1186/1745-6215-12-28

**Published:** 2011-02-02

**Authors:** David R Ellard, Stephanie JC Taylor, Suzanne Parsons, Margaret Thorogood

**Affiliations:** 1 University of Warwick, Clinical Trials Unit, Warwick Medical School, Gibbet Hill Road, Coventry, CV4 7AL, UK; 2 Queen Mary University of London, Barts & The London School of Medicine and Dentistry, Centre for Health Science, Institute of Health Science Education, Abernethy Building, 2 Newark Street, London E1 2AT, UK; 3 Picker Institute Europe, Kingsmead House, Oxpens Road, Oxford, OX1 1RX, UK; 4 University of Warwick, Warwick Medical School, Gibbet Hill Road, Coventry, CV4 7AL, UK

## Abstract

**Background:**

The OPERA trial is large cluster randomised trial testing a physical activity intervention to address depression amongst people living in nursing and residential homes for older people. A process evaluation was commissioned alongside the trial and we report the protocol for this process evaluation. Challenges included the cognitive and physical ability of the participants, the need to respect the privacy of all home residents, including study non-participants, and the physical structure of the homes. Evaluation activity had to be organised around the structured timetable of homes, leaving limited opportunities for data collection. The aims of this process evaluation are to provide findings that will assist in the interpretation of the clinical trial results, and to inform potential implementation of the physical activity intervention on a wider scale.

**Methods/design:**

Quantitative data on recruitment of homes and individuals is being collected. For homes in the intervention arm, data on dose and fidelity of the intervention delivered; including individual rates of participation in exercise classes are collected. In the control homes, uptake and delivery of depression awareness training is monitored. These data will be combined with qualitative data from an in-depth study of a purposive sample of eight homes (six intervention and two control).

**Discussion:**

Although process evaluations are increasingly funded alongside trials, it is still rare to see the findings published, and even rarer to see the protocol for such an evaluation published. Process evaluations have the potential to assist in interpreting and understanding trial results as well as informing future roll-outs of interventions. If such evaluations are funded they should also be reported and reviewed in a similar way to the trial outcome evaluation.

**Trial Registration:**

ISRCTN No: ISRCTN43769277

## Background

The OPERA trial (Older People's Exercise intervention in Residential and nursing Accommodation) is a cluster randomised controlled trial to evaluate the effect of an intervention to promote physical activity in reducing depression amongst residential and nursing homes residents. The protocol for the trial has been described in detail [1]. Briefly, the trial involves comparison of a control intervention to increase awareness of depression amongst staff in 77 residential and nursing homes with an active intervention which includes the same training in depression awareness, but also includes a whole-home physical activation programme, supported by a twice-weekly physiotherapist-led exercise class. The trial was commissioned the NHS Health Technology Assessment Programme, and a process evaluation was commissioned alongside the trial. In this paper we describe the protocol for the process evaluation.

We faced a number of challenges in designing the protocol for the process evaluation of this trial. We are working with a vulnerable population with varying degrees of cognitive and physical abilities, and must be careful to be inclusive in our approach to ensure that our findings reflect the experiences of the whole population of the care homes.

There are currently approximately 19,000 residential and nursing homes for older adults in England with a total capacity of 441,000 places [2]. Six out of ten places are in residential homes, the remainder in nursing homes [2]. Residential homes traditionally cater for less dependent residents than nursing homes and are typically staffed by social, as opposed to health care, personnel. As residents age, their health needs inevitably increase, leading to considerable overlap in the nursing care needs and dependency of residents in the two types of home [2]. Care homes are not part of the National Health Service, and owners include non-government organisations (NGOs), the private sector and local authorities; with some providers owning a single home and others owning a large number of homes in different geographical areas. Some homes are in converted buildings whilst others occupy purpose built premises. In many homes space to carry out interviews in privacy is limited. Researchers must also be aware that care homes are the homes of the residents and their right to privacy in their own home must be respected. This is true for those residents participating in the trial, but even more important with regard to non-participants. A care-home may agree to take part in the study, but not all residents will consent to participate (i.e. consent to provide baseline and follow up data from the study), although all residents who agree to an assessment by the OPERA physiotherapist will be able to attend exercise groups in the intervention homes. We needed to set up operating procedures that protected residents' privacy.

A normal day in a care home is structured with set times for meals, breaks, activities, and drug rounds which can leave little time for the researcher to interview or engage with the residents. During a pilot study, we found that there was often a narrow window of opportunity to engage in individual interviews. Residents tend to be more receptive in the mornings but this can clash with activities in the home. Even with morning interviews, residents are sometimes asleep at the time arranged for the interview. Moreover, many of the residents have a degree of cognitive impairment [3] limiting their ability to participate in research. Working in a care home as a researcher requires considerable organisational flexibility.

We aimed to include data gained from non-participant observations in a sample of homes but had to be flexible about how observations were carried out. We found that it was impossible to maintain non-participant observer status in this setting. The research fellow (DE) was inevitably drawn into the activities in the home, and residents became familiar with the researcher, and expected him to interact with them socially. Cohen and Crabtree [4] argue that participant and non participant observation complement each other. Observation aims to develop a rich understanding of a situation or setting and the behaviour of participants in that setting [4]. We decided that both non-participant and participant observation would be used.

Just as the design of a randomised trial is underpinned by a considerable theoretical literature, so the design of a process evaluation should be similarly underpinned. It is also important to clarify the theory that underlies the intervention being tested, because this will identify important processes to consider in the evaluation [5]. In our development of this process evaluation protocol, we have used the Theory of Change, [6-8] to identify the causal processes through which change comes about as a result of a programme's strategies and action [6], and have based the evaluation on the seven key components of process evaluation proposed by Steckler & Linnan [5], which are: context, reach, dose delivered, dose received, fidelity, implementation and recruitment. In addition, we decided that it was important to understand the views and opinions of participants, including residents of the homes, carers in the homes and members of the intervention team. Their experiences, their attitudes to the intervention and how they think the intervention could be improved will play an important part in interpreting the outcome data and developing consequent policy.

The process evaluation will use both quantitative and qualitative data. Although still poorly understood, mixed methods research is increasingly used in health services research and evaluation [9,10]. In process evaluation studies such methods assist the process of exploring apparent discrepancies between findings [11,12]. Integrating quantitative and qualitative methodologies into a single study requires careful planning from the outset [13]. Considerations include the priority given to a particular methodological approach and how other methods will complement that. The principal data collection method should be that which collects the data that is most important to the project's goals, while a complementary method offers strengths that add to the research design's overall ability to meet the project's goals [13]. In this study the principal data collection method is quantitative. The qualitative data will complement and illuminate the quantitative data but will only be collected from a small purposive sample of the homes (the case-study homes).

### Ethical Issues

Recruitment is an important component of the process evaluation and both the recruitment of homes (which are the units of randomisation) and the recruitment of participants are being evaluated. This population presents us with important ethical considerations. Many of the residents who are the potential recruits to this study will have some level of dementia and may not be able to make an informed decision to consent to participate in the research. Recruiting staff are required to make an assessment of residents prior to formally seeking their consent and if, in their judgement, they are not able to give consent then the next of kin will be contacted and asked if they would be willing to give informed assent.

As this is a pragmatic trial, it is important that as many residents as possible, with a wide range of cognitive abilities, are recruited to participate, so the consent/assent procedures are important. Observation of the consent process in the pilot study and a focus group with the recruiting staff revealed that residents were finding the consent process very long-winded and difficult to grasp. Discussions with the trial management team led to shortening of the consent form, but it remains an issue. Obtaining consent for the process evaluation interviews adds another layer of complication. In case study homes residents who are invited to be interviewed are taken through a separate consent process, and only residents who have consented (not assented) to provide data for the main OPERA study are eligible. This severely limits the available participants in some homes where many residents have cognitive impairments.

Not all residents in a home will consent/assent to participate in the trial. It is inevitable therefore that observation, such as home activity sweeps or observation of exercise classes, undertaken for the process evaluation will include non-participants. To preserve the privacy and rights of non-participants we will not identify any individuals in records of activity sweeps and exercise classes, and no researcher will go into any resident's bedrooms (unless specifically invited without prompting by the resident concerned)

## Methods/Design

The evaluation will support quantitative data collected from all homes with in-depth study of eight purposively selected homes. This will enable us to place homes in context, quantify the dose delivered, the dose received and the extent to which the target population are participating in the intervention (including recruitment rates for homes and individuals).

### Aims

• To provide findings that will assist in the interpretation of the clinical trial results;

• To inform potential implementation of the physical activity intervention on a wider scale.

### Objectives

• Carry out in-depth interviews with care managers, care staff, patients and carers in a purposive sample of the study sites;

• Observe the consent process, the exercise programme delivery and the depression awareness training;

• Document the RNH environment in case study homes and describe any changes observed;

• Collect quantitative data on number of patient/residents approached to take part in the exercise programme and the attendance at each session. Data will also be collected about the physiotherapists used and the number of sessions and homes that they work within;

• Collate and interpret all results and provide descriptive information and statistical analysis;

• Produce a process evaluation report and disseminate it through papers, articles and presentations.

### Methods

Date on the homes is collected at the outset of the study by the recruiting nurse and recorded on a pre-designed Case Report Forms (CRF) and entered into the trial database. Additional information from the Care Quality Commission website is extracted and entered into the database. The PE team monitors data collection regularly to ensure that the data needed for the process evaluation is good quality. Table 1 shows summary of the data collected from care-homes.

**Table 1:** Data collected from care-homes

Data	Source
Number of homes overall;	Home Recruitment log (number approached, declined)

Size of home (including number beds and actual occupancy);	Initial CRF^1^, CQC^2 ^report

Type of home in terms of specialty (e.g. Specialist or not);	Initial CRF, CQC report

Type of home in terms of funding (e.g. independent, charity, group or council/PCT run);	Initial CRF, CQC report

Level of activity already within home (classes, trips?)	Initial CRF

Facilities available within the home - accessible garden, exercise studio, ballroom, rooms for communal activities?	Initial CRF, CQC report, physiotherapist data

^1^Case Report Form, Data collection forms; ^2 ^Care Quality Commission reports obtained from website.

The staff in the homes are crucial participants in this intervention in both the control and active-intervention homes. Descriptive data regarding staffing will be collected for each home (see Table 2). In all homes, the physiotherapist or research nurse who delivers the depression awareness session record the number of staff attending and any additional training needs (for example staff who are unable to attend). At the end of the depression awareness training, evaluation forms are handed out asking staff to rate the session for delivery, understanding and usefulness. These are returned to the OPERA office. A second individualised questionnaire, with each member of staff's name added from register of training, is despatched to staff via the homes approximately four weeks after the training asking participants to assess how useful they have found the information they received.

**Table 2:** Data collected on care-home staff

Data	Source
Number (Day/Night), grade, vacancies, qualifications and number/type of ancillary staff	Initial home data CRF

Numbers trained (depression awareness/activity awareness), Attendance;	Attendance register (number only)

Number of training sessions run;	Attendance register (number only)

Satisfaction (with training/programme).	Evaluation form

Recruiting nurses complete a CRF for all the residents who have consented to participate in the Trial and for all residents where a next-of -kin has given assent. Table 3 provides a summary of the information that will be collected on individual residents.

**Table 3:** Data collected on residents

Data	Source
Numbers approached;	Recorded by recruiting nurse

Type of consent (personal or third party);	Recorded by recruiting nurse

Numbers agreeing to take part;	Recorded by recruiting nurse and in study folders

Drop-outs, adverse events or other attrition;	Collected three-monthly via CRF by recruiting nurse

Attendance on the exercise programme.	Attendance register

#### Case Study Homes

Qualitative data will complement and illuminate the quantitative data but is only collected from a small purposive sample of eight Case Study homes. Each case-study home is subject to in-depth study providing rich data. The main sampling criterion for these homes is ownership of the homes (see Table 4) and a secondary criterion in the intervention homes is homes being served by different physiotherapists delivering the intervention. There are equal numbers of homes from the two study areas. All homes recruited are informed about the process evaluation and the possibility that they will be asked to take part as a 'case study' home. Recruitment of case study homes takes place after randomisation, and is staggered over time, to allow enough time for the research team to develop a rapport with the home and gather required data.

**Table 4:** Case study home inclusion matrix

	Type of homes			
**Area**	**A**	**B**	**C**	**D**
Coventry and Warwickshire	1	1	1	1
North East London	1	1	1	1

A = Independent homes (< 6 homes in chain), B = Charity (non-profit) C = Nursing Homes D = Control Homes

Data is collected on three occasions; baseline, at six months and at the end of the study (12-months). Each data collection occasion takes around four days, spread over several weeks. Baseline face-to-face or telephone interviews are carried out soon after recruitment to the study and before OPERA interventions are implemented. Follow-up interviews with some of the key informants interviewed at baseline take place around six-months after implementation and at the end of the study.

The process of obtaining consent from participants is observed on a number of occasions in each of the homes (up to three participants in each home). This includes observing the process of consent when this is being given by a third party due to the cognitive impairment of the participant. Questions from participants are noted as are any areas of concern raised by the participant or the person carrying out the consent process.

Participants from each of the case study homes are invited to take part in semi-structured interviews, lasting no longer than 30 minutes, at a time and place that suits them. Interviews with next of kin or relatives may be carried out via telephone for the convenience of the respondents. Participants to be interviewed include:

• Care Home Manager (plus Group manager if appropriate)

• Care Home Staff (up to three per home)

• Care home Residents (up to three per home)

• Residents relatives/next-of-kin (up to two per home)

• Physiotherapist (involved in the intervention in that home)

Inclusion Criteria for residents to take part in interviews are:

• Ability to understand and communicate in spoken English (In particular residents);

• Has given consent to participate in the OPERA main study;

Exclusion Criteria are:

• Severe cognitive impairment (i.e. not competent to consent);

• Severe problems communicating.

Interviews at baseline explore life in the home and the current levels of activity, staff/resident interactions and the process of consent to the OPERA trial. Follow-up interviews explore in more depth perceptions about the home and its levels of activity and, in intervention homes, perceptions of the activity programme and its impact, and at the last interview, feelings about the programme being withdrawn at the end of the study.

Additional information is sought from the care home managers/group managers about their reasons for taking part, their feelings about the whole home study and their beliefs about the potential long-term changes in the home as a result of the intervention.

Non-participant and participant observations by the Research Fellow (DE) take place in the case study homes. Observations form a substantial part of the evaluation and include the home environment, levels of activity and staff/resident interactions. On each visit to a home the Research Fellow makes field notes, including noting time of day, what residents are doing, any activities, staff interactions, general ethos and any contacts made whilst there. In intervention homes it also includes how OPERA 'exercise sessions' fit in with the daily routines of the home and how these are received/perceived. In addition, there is observation of the 'processes' and procedures of the OPERA whole-home intervention. Observations will include:

• Observation of the home environment;

• Observation of the consent/assent process;

• Delivery of the Depression Awareness Training in control homes and Depression Awareness & Activity Training in intervention homes;

• Delivery of the exercise sessions;

• 'Activity'.

As described above, staff in all of the homes (intervention and control) receive a short training session on depression awareness. Intervention homes receive additional information about activity. At least one of these training sessions will be observed in each of the case study homes. This is in addition to the questionnaire distributed to staff in all the Trial homes, as mentioned above. Key elements that are noted include:

• Staff response to these sessions;

• How confident the presenter is in engaging/adapting to participants needs;

• The environment used for the training (room, noise, time to participate, distractions);

• Timing (how-long);

• Level of interest (questions?).

In the six case-study intervention homes, The Research Fellow observes several exercise classes in the early stages of their introduction, at about the mid-point and at the end of the 12-months. Observation includes:

• Ambience/atmosphere

○ Does it feel relaxed and easy?

○ How does the session fit into the day

■ e.g. is it close to lunch or tea-time

• How are participants reacting

○ Are they joining in?

○ Are they happy?

○ Are they dissenting?

○ Interaction with session leader

• How are the session leaders performing

○ With confidence?

○ Adapting to audience?

○ Listening?

○ Working within defined manual?

• Setting

○ Setting up

○ Getting to classes (residents)

○ Getting away from the class (residents)

○ Room?

○ Distractions?

○ Involvement of care home staff?

• Timing, how long was the session?

○ What happens when the class ends?

The observational instrument of activity and well-being Behaviour Category Codes (BCC) [14,15] is used in structured observation sweeps of all case study homes at baseline and follow-ups. This involves the Research Fellow completing a checklist of where residents are what they are doing and what care-staff are doing at regular intervals in the day. Briefly, the observations are carried out in the following way:

1. Observational data sweeps occur every fifteen minutes, for a 90 minute period;

2. No more than three hours of observation should occur in any one day;

3. Observation periods reflect the daily life in the home: Recommended time periods: 10 am-11.30 am, 12 pm-1.30 pm, 2 pm-3.30 pm, 4 pm-5.30 pm, 6 pm-7.30 pm (7.5 hours total).

Different activities are categorised e.g. (DE insert some on the lower level categories), and grouped e.g. "active social interaction", "recreational activity". Sweeps only register the ratio of residents in each public area exhibiting a particular behaviour; no individuals will be identified. No private areas, such as bedrooms are included.

#### Qualitative Data Analysis

Interviews are digitally recorded, subject to permission of each participant, and where appropriate, are transcribed verbatim after anonymisation. The recordings are stored in a secure digital environment. The software package NVivo 7 [16] will be used to facilitate analysis. Researcher bias will be minimised through regular crosschecking of data and findings by the members of Research Team. In addition, transcripts will be returned to participants (where appropriate) providing them with the opportunity to check the transcripts for accuracy and authenticity and to offer any subsequent reflections. Anonymised quotations will be used where possible as exemplars of key points in the writing up of these data.

#### Quantitative Data Analysis

Quantitative data will be analysed using the statistical package PASW Statistics [17]. Descriptive statistics will be generated and comparisons made between homes or types of homes.

Data analyses are ongoing and process evaluation data will be analysed independently of the main study, before the two data sets are combined [11]. Data are extracted and fed back to and reviewed by the Process Evaluation Team at regular intervals to ensure good quality data. Themes emerging from qualitative data are also discussed and refined by the Process Evaluation Team. A sample of 10% of transcripts will be coded by another member of the team to ensure reliability and validity.

#### Ethics

The OPERA trial and its process evaluation has ethical approval from the UK National Research Ethics Service (Ref. no. 07/Q0505/56)

## Discussion

In preparation of this study, we researched the process evaluation of trials. We did not find very many examples of independent publications of the outcome of process evaluations and did not find any published protocols. Nevertheless, it is becoming increasingly common to fund process evaluation and such an evaluation is now accepted as good practice in randomised controlled trials [11]. Process evaluation should be a fundamental part of trial development, particularly in the complex trials of behaviour change such as this. Such evaluations provide insights into how the trial intervention was delivered and into how participants experienced receiving either the intervention or the control treatment. These insights may be crucial to the interpretation of the trial results and the further development of the intervention being tested. If process evaluations are funded and undertaken, then we believe that both the protocols for process evaluation and the findings of such evaluations should be published. This will have to dual advantages of providing more information to appropriately interpret the findings of randomised trials and also encouraging the improvement and development of process evaluation protocols and reporting.

In common with many emerging methods of enquiry, process evaluation tends to attract minimal funding. This may reduce the quality of the data provided. It is important to maintain a distance between the process evaluation team and the main study team [11]. In our case the limited funding meant that such a separation was out of the question and all members of the process evaluation team also had roles in the main trial. However, we have endeavoured to maintain a separation between the process evaluation activities and findings and those of the team evaluating effectiveness. Analysing process data independent of the outcome data [11], will allow for the generation of hypotheses or research questions that can be tested in statistical analyses (at the end of the trial and process evaluation) integrating process and outcome data. For example, data from the process evaluation can be used to explore the individual context of each site. Sub-grouping sites based on context may help to explain variations in the effectiveness of the intervention.

## Competing interests

The authors declare that they have no competing interests.

## Authors' contributions

DE participated in the design and day-to-day management of the process evaluation and drafted the manuscript. ST, SP and MT participated in the design of the process evaluation and drafting of the manuscript. All authors contributed to refinement of the study protocol and approved the final manuscript.
